# Workaholism and Technostress During the COVID-19 Emergency: The Crucial Role of the Leaders on Remote Working

**DOI:** 10.3389/fpsyg.2020.620310

**Published:** 2020-12-23

**Authors:** Paola Spagnoli, Monica Molino, Danila Molinaro, Maria Luisa Giancaspro, Amelia Manuti, Chiara Ghislieri

**Affiliations:** ^1^Department of Psychology, University of Campania Luigi Vanvitelli, Caserta, Italy; ^2^Department of Psychology, University of Turin, Turin, Italy; ^3^Department of Education, Psychology, Communication, University of Bari Aldo Moro, Bari, Italy

**Keywords:** technostress, workaholism, authoritarian leadership, conditional process analysis, remote working

## Abstract

Although remote working can involve positive outcomes both for employees and organizations, in the case of the sudden and forced remote working situation that came into place during the COVID-19 crisis there have also been reports of negative aspects, one of which is technostress. In this context of crisis, leadership is crucial in sustainably managing and supporting employees, especially employees with workaholic tendencies who are more prone to developing negative work and health outcomes. However, while research on the role of the positive aspects of leadership during crises does exist, the negative aspects of leadership during the COVID-19 crisis have not yet been studied. The present study aimed to explore the role of authoritarian leadership in a sample of 339 administrative university employees who worked either completely from home or from home and the workplace. The study examined the moderating effect of a manager on this relationship and the connections between workaholism and technostress through conditional process analysis. Results pointed out that high authoritarian leadership had an enhancing effect, whereas low authoritarian leadership had a protective effect on the relationship between workaholism and technostress, only in the group of complete remote workers. Thus, authoritarian leadership should be avoided and training leaders to be aware of its effect appears to be essential. Limitations, future directions for the study, and practical implications are also discussed.

## Introduction

Due to the COVID-19 lockdown, administrative staff at universities as well as many other service employees suddenly shifted from traditional working modalities to remote working. Consequently, one of the most important challenges for university management was the creation of a virtual environment in which employees could continue working. Remote working can have some positive outcomes, such as improved performance, cutting the costs of “home-work-home” traveling, saving time, and organizational resources, and increasing employee satisfaction (Barbuto et al., 2020; Thulin et al., 2020), however, some negative consequences have also been highlighted, particularly in relation to wellbeing, and it can cause stress, discomfort, and anxiety due to the constant use of the Internet, email, instant messaging, and smartphones (Salanova et al., 2013). In a recent contribution, Molino et al. (2020) reported on the effects of technology use on wellbeing during COVID-19 mandatory remote working, or technostress, namely “the stress that users experience as a result of application multitasking, constant connectivity, information overload, frequent system upgrades and consequent uncertainty, continual relearning and consequent job-related insecurities, and technical problems associated with the organizational use of ICT” (Tarafdar et al., 2010, pp. 304–305). Although these wellbeing costs might affect some remote workers, we believe that they might have specifically caused trouble for workaholic workers, namely “persons whose need for work has become so excessive that it creates noticeable disturbance or interference with (their) bodily health, personal happiness, and interpersonal relations, and with (their) smooth social functioning” (Oates, 1971, p. 4). The effects of wellbeing on a sudden change in working processes might have been particularly detrimental for workers who are addicted to their job, since they might have perceived the change as hindering their usual job routine, with an amplified feeling of guilt, anger, anxiety, and frustration, and, therefore, in general, a more stressful experience.

In this context of change and crisis, leadership plays a crucial role (Bartsch et al., 2020; Bodolica and Spraggon, 2020). Research examining the role of leadership behavior in the context of planned organizational change is well established (e.g., Oreg and Berson, 2019; Sverdlik et al., 2020), and more recent studies in response to the pandemic crisis have focused on “being a smart leader” or an “e-leader” (Cortellazzo et al., 2019; Iannotta et al., 2020) while also at the same time, being an effective leader (Bartsch et al., 2020). However, few leadership studies discuss ineffective leadership behaviors in the context of rapid and unpredictable organizational transformation like that of the COVID-19 pandemic. It is likely that, since they are deprived of forms of physical control in the workplace, leaders might exaggerate the authoritative style they use to control the performance of employees. This can manifest as an invasion into the private life of employees, relying upon the situation of being “always-on” that is created by the constant use of communication technologies when remote working.

In line with these speculations, with special reference to the peculiar working conditions imposed by the spread of COVID-19, which are mostly based on mandatory remote work, and the hierarchic work organization of the academic context, this study aimed to investigate if and to what extent authoritarian leadership behaviors could be a moderator of the relationship between workaholism and technostress in employees.

### Workaholism and Technostress

Technostress is defined as “the phenomenon of stress experienced by end users in organizations as a result of their use of ICTs” (Ragu-Nathan et al., 2008, pp. 417–418). The symptoms related to technostress include anxiety, behavioral strain, feelings of exhaustion, mental fatigue, poor concentration, physical diseases, and insomnia, while its main consequences are reduced productivity, job satisfaction, and organizational commitment and increased employee outcomes (e.g., absenteeism and turnover) (e.g., Tarafdar et al., 2010; Ayyagari et al., 2011; La Torre et al., 2019). The use of ICTs might challenge employees by creating a variety of stressors, including information overload, role ambiguity, job insecurity (Fenner and Renn, 2010; Grant et al., 2013), the intensity of teleworking (Suh and Lee, 2017), high quantities of e-mails, poor e-mail quality (Brown et al., 2014), and frequent interruptions during work (Ninaus et al., 2015).

A widely accepted scientific classification of the creators of technostress is proposed by Tarafdar et al. (2007) who used a transactional approach to describe five techno-stressors: (1) techno-overload (ICTs increase the pace and volume of work and induce users to work faster and longer); (2) techno-invasion (ICTs invade personal life and blur boundaries between work and private domains); (3) techno-complexity (ICTs’ complexity leads to feelings of incompetence); (4) techno-insecurity (workers feel threatened by job loss to automation or other people who have a better knowledge of ICT); and, (5) techno-uncertainty (continuous changes or upgrades in ICTs that generate ambiguity and disturb users). Moreover, ICTs and Internet connection enable constant availability and 24/7 access to work. The increased use of ICTs has engendered expectations about workers being always available and working faster and better (World Health Organization, 2005). In light of this, it is interesting to investigate the interaction between technostress creators and work addiction.

Workaholism is the tendency to work excessively hard and to be obsessed with work. Thus, it consists of two main dimensions: working excessively (tendency to allocate remarkably much time to work than to other life activities and to work beyond what is reasonably expected) and working compulsively (a strong inner drive to work hard and to think about work, even when not working) (Schaufeli et al., 2008). Workaholics invest a lot of time and energy in their work, without respecting any boundaries between work and private lives. They also work in the evening and at the weekend, at the cost of other private and family activities and relationships.

Previous studies have found a positive relationship between workaholism and job stress and burnout (Taris et al., 2005; Clark et al., 2016a; Andreassen et al., 2018a), psychophysics strain (Falco et al., 2013), low sleep quality, and daytime sleepiness (Spagnoli et al., 2019), anxiety/insomnia, somatic symptoms, and social dysfunction (Andreassen et al., 2018b), and work-family conflict (Bonebright et al., 2000; Taris et al., 2005; Bakker et al., 2009). Although the determinants of workaholism are not questioned here, a recent meta-analysis has shown that it is linked to both personal and organizational factors (Clark et al., 2016a), and, despite there being a lack of evidence on the relationship between remote working and workaholism, we believe it is likely that the absence of defined boundaries between work and life could represent a risk factor.

To date, the relationship between workaholism and ICTs has been primarily referred to as the phenomenon of techno-addiction (an uncontrollable “have to” pressure paired with anxiety when not using ICTs, which leads to the use of them for long periods in an excessive way) (Salanova et al., 2013) or to the fact that being a workaholic could lead to intensive smartphone use (Spagnoli et al., 2019). Nevertheless, since workaholism entails a combination of concern and a craving to always stay connected to work, it is interesting to observe its relationship with techno-stressors and, more specifically, to investigate whether workaholism might increase the risk of technostress.

*Hypothesis 1*: *Workaholism is positively related to technostress*.

### The (Moderated) Moderating Role of Authoritarian Leadership in the Relationship Between Workaholism and Technostress

The leadership construct has attracted scientific attention due to the positive impact it exerts within an organizational context. However, to date, very few studies have focused on the potentially harmful effects of leadership behaviors or the negative impact that misconduct can have both on individual and organizational outcomes (e.g., Pelletier, 2010; Ghislieri and Gatti, 2012; Ghislieri et al., 2019). Studies that do address the negative impact of leadership styles mostly refer to the concept of authoritarian leadership, stemming from the early experimental studies by Lewin et al. (1939). This style is usually characterized by behaviors that centralize decision-making and exert power and control over subordinates without any consideration of their contribution or productivity (Sauer, 2011). Authoritarian behaviors might include giving orders to followers, telling them what to do, and making decisions in a unilateral way (De Hoogh and Den Hartog, 2009).

The basis of authoritarian power is derived from the opportunities created by the leader’s position in the organization, with control over resources and rewards (Cheng et al., 2004). Yet, this form of “toxic” leadership (Schmidt, 2008) could be concretely enacted by a broad variety of negative behaviors (Pelletier, 2010) such as intimidating, bullying, manipulation, micromanaging, and engaging in abusive or unethical behavior. Several scientific studies have documented that authoritarian leadership negatively affects subordinates in terms, for example, of increasing spontaneous aggression and hostile behaviors, decreasing job satisfaction, and trust in management (see Bass and Bass, 2008 for a comprehensive review). Early social psychology studies showed that authoritarian leadership tends to increase spontaneous aggression and hostile behavior (Lewin et al., 1939). More recently, studies in the field of management sciences have suggested that it also harms the attitudes and behaviors of subordinates, including job satisfaction (Smither, 1993), organization-based self-efficacy (Chan et al., 2013), trust in management (Chen et al., 2014), interactional justice (Wu et al., 2012), organizational voice behaviors (Li and Sun, 2015), task performance, and conscientious behavior (Wang et al., 2013). Contingency theories have affirmed that specific contextual factors such as role ambiguity and uncertainties (Rast et al., 2013) may increase the effectiveness of authoritarian leadership (Yukl, 2011), as well as the dependence and compliance of followers (Chou et al., 2015).

The present study focused on the moderating role played by an authoritarian leadership style on the relationship between an employee’s attitude toward their job, namely their perception of workaholism and technostress. Accordingly, the study was conducted in an academic context and involved university administrative staff during the COVID-19 pandemic, where all participants were forced to working remotely and therefore were supposed to be exposed to increased use of technology. Yet, following studies conducted in public management, affirming the difference between public and private organizations in leadership style (Anderson and Anderson, 2010), the study assumed that the academic context could be characterized by the presence of an authoritarian leadership style, because public managers operate under a different set of organizational or procedural constraints compared to private managers. Accordingly, the organization of work within the public context seems to be attuned to the main components of authoritarian leadership (Farh and Cheng, 2000), which involve top-down communication, control information, and an underestimation of subordinate competence.

This study explored the idea that remote working is a condition that could deprive employees of physical controls and therefore, leaders might exaggerate their authoritative style to control the performance of subordinates. This could manifest as an invasion of private life by relying upon the situation of being “always on” that is facilitated by communication technologies. On the other hand, employees might be pushed to work harder and compulsively to meet the demands of leaders and avoid retaliation, punishment, and negative feedback (Molino et al., 2019), thus increasing technostress.

In position papers about the research needs in COVID-19 emergency, the experts recommendation suggest to deepen the role of the leadership (Kniffin et al., 2020). Even though many studies focus on the “light” side of leadership, more and more scholars have recently outlined the “darker” aspects of leadership, particularly based upon several informal reports by workers (Molino et al., 2019) and, the stress dynamics of work. Our study is rooted in this perspective and, between the different facets of the “toxic” leadership, took into account authoritarian leadership concerning the central position of control in this expression of leadership (Cheng et al., 2004), which is challenged in the context of remote work.

Authoritarian leadership may have a moderating role in the relationship between workaholism and technostress, following the self-determination theory (SDT) by Deci and Ryan (2000). In line with previous studies (e.g., Chu, 2014), authoritarian leadership insists on control and, places people in a state of powerlessness, a condition that can exacerbate the effect of workaholism on technostress. The process by which workaholism is associated with negative outcomes can be related to the quality of motivation and action, as Van den Broeck et al. (2011) have highlighted. Through actions that limit self-determination, authoritarian leadership further undermines the autonomy of workers through forms of control that, in remote work, pass through ICT, enhancing the effect of workaholism on a negative result such as technostress.

The current study took place soon after the COVID-19 lockdown and some of the university employees decided to keep working remotely. Other employees started to work in a “hybrid” way, involving some days at home and some days in the workplace. We believe that the negative effect of authoritarian leadership could have been stronger for employees who work remotely full time. Given the distance, a lack of live contact and communication, an authoritarian leadership style might have been perceived as more incisive and intrusive with more negative outcomes for those who worked remotely. Thus, we put forward the following hypotheses:

*Hypothesis 2*: *Higher levels of authoritarian leadership and workaholism are positively related to technostress.*
Hypothesis 3: Higher levels of workaholism and lower levels of authoritarian leadership decrease technostress.
Hypothesis 4: The effect of authoritarian leadership is stronger for the employees working in a completely remote condition.

We tested hypotheses controlling the effect of personal data, with a focus on gender. Scientific literature showed results on the relationship between gender and technostress that are contrasting and scarce. Some contributions have outlined that men tend to show more positive attitudes toward technology, with less self-control and that they are more prone to developing problematic behaviors than women, especially for agentic purposes (Lee et al., 2014). Conversely, other studies have highlighted that women are less inclined to use technology in the workplace (Venkatesh and Morris, 2000), that they sometimes find it complicated, and develop higher anxiety and phobia (Whitley, 1997).

## Methods

### Participants and Procedure

Data were collected through an online self-report questionnaire within a project that involved consulting the technical-administrative staff for the introduction of new management policies related to remote work during the COVID-19 emergency in July 2020. Participants had 2 weeks to answer the questionnaire, which took about 15 min to complete. The link for filling the online questionnaire was sent to 867 employees of an Italian University. At the end of the questionnaire, administration data were available for 359 individuals. Then 20 participants were excluded due to missing values. Thus, 339 employees were involved in the study. They were 46.6% male and 53.4% female. Age ranged from 22 to 70 years old (Mean = 48.43; St. Dev. = 9.71). Education was: 59% bachelor or master degree; 38.6% high school; and 2.4% middle school. Regarding their role, 34% held a position of responsibility and most of them (85.3%) declared a tenure of more than 10 years. More than half of them (52.7%) worked partially remotely, alternating days of work at home and days of work in the workplace, whereas the rest (47.3%) always worked remotely from home.

### Ethics Statement

This study was in accordance with the standards of national laws on data treatment as followed by the University of Campania “Luigi Vanvitelli,” which is part of the University of Torino and University of Bari (Italy). Since there was no medical treatment or other procedures that could cause psychological or social discomfort to participants, who were all anonymous adult healthy subjects, additional ethical approval was not required. The research was conducted in line with the Helsinki Declaration (World Medical Association, 2001), as well as the data protection regulation of Italy (Legislative Decree No. 196/2003). Participation in the study was voluntary and not rewarded, and data collection and analysis were anonymous. A covering letter, attached to the questionnaire, provided information about the aims of the study, guarantees about anonymity, voluntary participation, data treatment, and instructions for filling out the questionnaire. When agreeing to fill out the questionnaire, all study participants provided their informed consent.

### Measures

#### Workaholism

Workaholism was measured by the 10-item version of the Dutch Work Addiction Scale (DUWAS), which was adapted and validated in Italian (Balducci et al., 2015). The DUWAS investigates the respondent’s feelings about their work, which reflects the two components of workaholism (i.e., working compulsively, WC, and working excessively, WE). Example items are the following: “I feel that there’s something inside me that drives me to work hard” (WC) and “I stay busy and keep many irons in the fire” (WE). Responses were given on a 6-point scale varying from 1 (“Never or almost never”) to 6 (“Almost always or always”). Cronbach’s alpha is 0.85.

#### Authoritarian Leadership

Authoritarian Leadership was measured by the six-items from the Toxic Leadership Scale (Schmidt, 2008). Participants were asked to respond about the occurrence of leader typical authoritarian behaviors in recent weeks. An example item is the following: “They are inflexible when it comes to organizational policies, even in special circumstances.” Responses were given on a 6-point scale ranging from 1 (“Never”) to 6 (“Always”). Cronbach’s alpha is 0.81.

#### Technostress

Technostress was measured by the 9-items version of the Technostress Creator Scale (TCS -Ragu-Nathan et al., 2008), which was adapted and translated into Italian by Molino et al. (2020), with three items for techno-overload, three items for techno-invasion, and three items for techno-complexity. In this study, we considered these three dimensions because of their relevance to the current scenario, where the increase of technology use, due to remote working leads workers to experience overload, an intrusion of work into their private life, and difficulties in managing complex technologies. An example is: “I do not find enough time to study and upgrade my technology skills.” Responses were given on a 6-point scale ranging from 1 (“Completely disagree”) to 6 (“Completely agree”). Cronbach’s alpha is 0.87.

### Data Analysis

**FIGURE 1:**
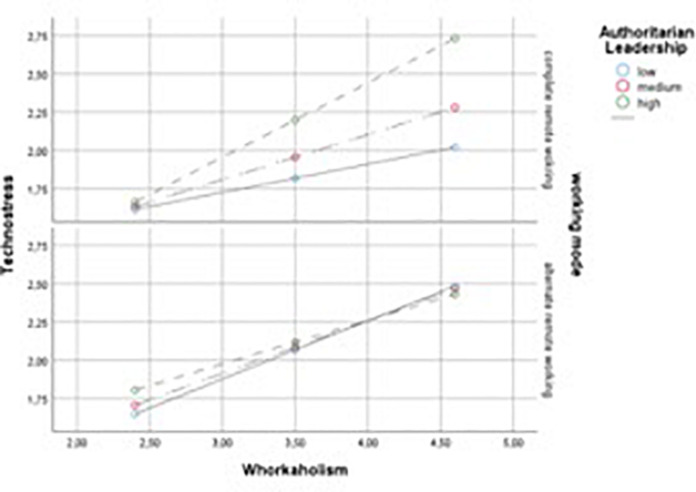
Plots for the moderated (moderated) analysis.

Zero-order correlations were used to examine the associations between variables. Reliability analysis was used to assess the internal consistencies of the scale. A series of ANOVAs were conducted to better examine the role of gender in the study variables. The hypotheses concerning direct and moderated effects were tested through conditional process analysis based on OLS regression using bootstrapping technique (Hayes, 2017), a non-parametric resampling procedure that does not assume normality extracted several thousand subsamples (5000, in our case) from a dataset. Through bootstrapping, the distribution of effects was empirically approximated and used for calculating confidence intervals. We tested a moderated moderation, where the direct effect of workaholism on technostress is moderated by authoritarian leadership, and the moderating effect of authoritarian leadership is, in turn, moderated by the dichotomous variables “working mode” (i.e., complete remote working/alternate remote working). The model examined in the current study is represented in Figure 1, it corresponds to the conceptual model number 3 of Hayes templates.

## Results

Before conducting the main analysis, we computed the risk for common method bias through the Harman single-factor test. The variance explained by the single factor, including all the observed variables, was only 23%. Thus, we concluded that the risk for common method variance was low.

Table 1 shows descriptive analysis and zero-order intercorrelations of the variables in the study. Results pointed out that workaholism positively and significantly correlated with both authoritarian leadership and technostress. We also ran a series of ANOVAs to better examine the role of gender in the study variables. Results pointed out that women had statistically significant higher scores on technostress (*F* = 4.57; *p* < 0.05), while no gender differences were detected for workaholism (*F* = 0.52; *p* = 0.47) and authoritarian leadership (*F* = 1.34; *p* = 0.25). In particular, the mean score for women on technostress was *M* = 2.18 (*SD* = 1.07), and for men, it was *M* = 1.95 (*SD* = 0.85). To better assess the hypothesized model we added gender as well as age to the tested model. Table 2 concerns the results of the conditional process analysis on technostress. Although both workaholism and authoritarian leadership seemed to not be significantly and directly related to technostress, the interaction between them was significantly related to it (*B* = 0.62, LLCI = 0.23, ULCI = 1.01). Moreover, the working mode (complete/alternate remote working) was not significantly related to technostress as well as the interactions between workaholism and working mode (complete/alternate remote working) and between authoritarian leadership and working mode (complete/alternate remote working). Finally, the interaction among workaholism, authoritarian leadership, and remote working was significantly related to technostress (*B* = −0.22, LLCI = −0.39, ULCI = −0.06).

**TABLE 1:** Descriptions and intercorrelations of the study variables.

	**Mean**	**St. Dev.**	**Gender**	**Age**	**Workaholism**	**Authoritarian leadership**
Age	48.29	10.06	–0.04			
Workaholism	3.5	1.05	0.04	0.06		
Authoritarian Leadership	2.3	1.01	0.06	−0.14**	0.21**	
Technostress	2.07	0.98	0.12*	0.18**	0.40**	0.18**

**p < 0.01; *p < 0.05. Gender was coded as 1 = men and 2 = women.

**TABLE 2:** Conditional process analysis on technostress.

**Variables**	**B**	**LLCI**	**ULCI**	***R*^2^**
Outcome: Technostress				0.25*
Workaholism	0.41	–0.04	0.87	
Authoritarian Leadership	0.56	0.07	1.04	
Workaholism * Authoritarian Leadership	0.55	0.17	0.93	
Working Mode (complete/alternate remote working)	0.06	–0.13	0.24	
Workaholism * Working Mode (complete/alternate remote working)	–0.03	–0.21	0.14	
Authoritarian Leadership * Working Mode (complete/alternate remote working)	–0.17	–0.36	0.01	
Workaholism * Authoritarian Leadership * Working Mode (complete/alternate remote working)	–0.19	–0.35	–0.03	
Gender	0.19	0.01	0.37	
Age	0.02	0.01	0.03	
**Moderated effect of workaholism on Technostress**				
Low authoritarian leadership/complete remote working	0.19	0.03	0.34	
Low authoritarian leadership/alternate remote working	0.34	0.15	0.52	
Medium authoritarian leadership/complete remote working	0.30	0.17	0.43	
Medium authoritarian leadership/alternate remote working	0.31	0.18	0.45	
High authoritarian leadership/complete remote working	0.48	0.33	0.63	
High authoritarian leadership/alternate remote working	0.28	0.11	0.44	

*p < 0.05.

**FIGURE 2:**
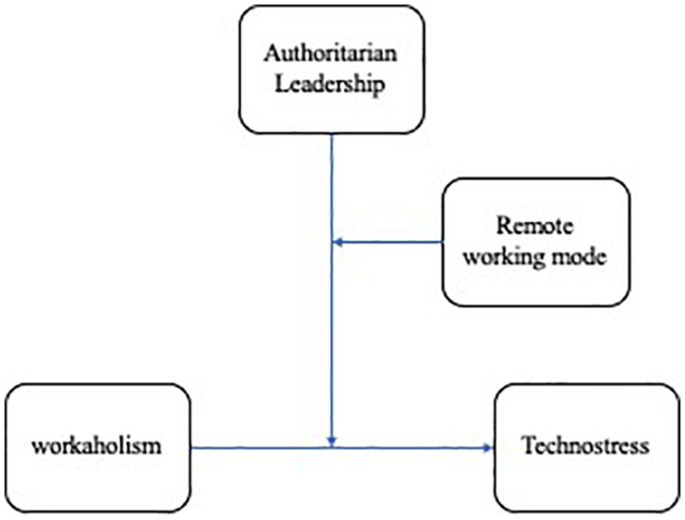
Conceptual model of the study.

Following Hayes (2017), the values of workaholism were observed at the 16th, 50th, and 84th percentile of authoritarian leadership. In the complete remote working plot displayed in Figure 2 when workaholism is high and authoritarian leadership is high, technostress is significantly higher than when authoritarian leadership is low. As far as the simple slopes are concerned, results pointed out that all the six simple slopes were statistically significant, with the highest effect for the combination of high levels of authoritarian leadership in the group of complete remote working (*B* = 0.51, LLCI = 0.36, ULCI = 0.67). However, a test of the conditional interaction of workaholism and authoritarian leadership at the two levels of working mode revealed that the positive effect (*B* = 0.17, *p* < 0.001) was significant only for the complete remote working mode, whereas was not significant for the alternate remote working mode (*B* = −0.06, *p* = 0.36). Thus, we concluded that high authoritarian leadership had an enhancing effect whereas low authoritarian leadership had a protective effect on the relationship between workaholism and technostress, but only in the group of complete remote workers.

## Discussion

The current study, based on the self-determination theory (Deci and Ryan, 2000), aimed to test if and to what extent an authoritarian leadership style might moderate the relationship between workaholism and technostress in a sample of university administrative staff who worked either totally or partially remotely during the COVID-19 emergency during summer 2020. Our hypotheses were supported and the interaction between workaholism and authoritarian leadership was significantly related to technostress. The effect of this interaction particularly concerned those employees who worked remotely full time. In particular, our study indicated that high levels of authoritarian leadership enhanced the positive relationship between workaholism and technostress and that it boosted the effect of workaholism on technostress, which was significantly higher than when the level of authoritarian leadership was low.

These results are in line with literature on the negative outcomes of authoritarian leadership (Bass and Bass, 2008) and supports the original assumption of this study, that authoritarian leadership might be harmful and enhance the technostress of employees with a compulsive work ethic. Moreover, the moderating effect was significant only for those who worked remotely. This could be, because the absolute distance between employees and their managers might exacerbate the perception of invasion or the leader’s unilateral decision-making. On the other hand, a leader’s behaviors toward workers who alternate between remote and office working might be or at least perceived by the workers as being less invasive. This situation could be more participatory in terms of the decision-making process, given that both the leader and the employee can meet at the workplace.

In terms of gender differences, the results confirmed that technostress was higher for women. These results are consistent with previous evidence (Whitley, 1997; Venkatesh and Morris, 2000; Lee et al., 2014). Men are generally involved in more complex and technology-based tasks, while women have fewer opportunities to develop technology confidence (Brussevich et al., 2018), also because of occupational segregation, which is particularly dominant in Italy and among university staff.

While these results provide meaningful research evidence and could have useful practical implications, they should be considered in light of the study’s limitations. This was a, cross-sectional study and data were self-reported. A longitudinal study would provide a more robust method of testing the study hypotheses, and a larger collection including multiple sources would strengthen results.

Moreover, recent literature has also emphasized the role of situational factors, for instance of the work context, in exacerbating workaholic behavior among employees prone to developing this compulsive behavior (e.g., Di Stefano and Gaudiino, 2019). The presence of a reciprocal relationship between technostress and workaholism should be addressed in future studies.

A further avenue for future research could also be an investigation of the impact of the behavior of workaholic behavior as conducive to work obsession among subordinates (Clark et al., 2016b) and the likelihood that other leadership styles may intensify the relationship between workaholism and technostress (e.g., transformational leadership), as suggested by prior research (Andreassen, 2014).

Future studies should also explore how work engagement, may exhibit a similar relationship to technostress and how a positive psychological relationship with one’s work might affect this situation. Engagement and workaholism are described in recent literature as different forms of heavy work investment, characterized by a high absorption in work (Snir and Harpaz, 2012). It is, therefore, reasonable to assume that engaged employees might also exhibit high levels of technostress, stemming from the blurred boundaries between work and private life due to the greater occurrence of remote working.

In the future, studies should investigate the role of gender in relation to technology-use and technostress in more detail. They could consider factors such as age, as according to Morris et al. (2005) gender differences are not relevant in young employees and the dimensions of specific types of technostress. Other recent studies have pointed out that there are higher levels of techno-complexity and techno-uncertainty in women, while men are more prone to techno-overload and techno-invasion (Marchiori et al., 2019).

The practical implications of this study are that organizations must monitor the risk of workaholism and any signs of technostress, through organizational analysis tools. This is particularly important during times of crisis when targeted investigations can be used to introduce immediate corrective measures, avoiding dangerous cycles of behavior. Training on psycho-social risks and the introduction of good practices relating to disconnection (during non-work times) are achievable preventive interventions. Other interventions could, include adequate forms of individualized psychological support.

As far as leadership roles are concerned, negative effects must be avoided during the selection and socialization phase, and they should be alert to the abusive and demanding behavior facilitated by a technology-based work environment, which violates employee privacy. Sometimes organizational cultures may induce or fuel these behaviors (even unintentionally) as managers are inclined to test the loyalty of subordinates through excessive requests and tele-pressure (Van Laethem et al., 2018). Training is a crucial way of reducing the impact of authoritarian leadership behaviors. A targeted training program is important in addressing specific forms of authoritarian relationships, enabling interventions in these relationships (Ghislieri and Gatti, 2012) and helping people to cope with abusive supervision (Harvey et al., 2007), whilst also helping organizations avoid negative authoritarian processes.

## Data Availability Statement

The raw data supporting the conclusion of this article will be made available by the authors, without undue reservation.

## Ethics Statement

Ethical review and approval was not required for the study on human participants in accordance with the Local Legislation and Institutional Requirements. The patients/participants provided their written informed consent to participate in this study.

## Author Contributions

PS: conceptualization, formal analysis, and project administration. PS and DM: methodology, software, and data curation. PS, MM, MG, AM, and CG: writing–original draft preparation and writing–review and editing. All authors contributed to the article and approved the submitted version.

## Conflict of Interest

The authors declare that the research was conducted in the absence of any commercial or financial relationships that could be construed as a potential conflict of interest.
